# Plasma C-Reactive Protein and Pentraxin-3 Reference Intervals During Normal Pregnancy

**DOI:** 10.3389/fimmu.2021.722118

**Published:** 2021-08-02

**Authors:** Lina Wirestam, Sofia Pihl, Muna Saleh, Jonas Wetterö, Christopher Sjöwall

**Affiliations:** ^1^Department of Biomedical and Clinical Sciences, Division of Inflammation and Infection/Rheumatology, Linköping University, Linköping, Sweden; ^2^Department of Obstetrics and Gynaecology, Linköping University, Linköping, Sweden; ^3^Department of Biomedical and Clinical Sciences, Linköping University, Linköping, Sweden

**Keywords:** C-reactive protein, pentraxin-3, pregnancy, reference interval (RI), biomarkers, acute phase protein, inflammation

## Abstract

Although several biomarkers are available to monitor the acute phase response, the short pentraxin C-reactive protein (CRP) is dominating in clinical practice. The long pentraxin 3 (PTX3) is structurally and functionally related to CRP, but not liver-derived. In addition, increased levels of PTX3 have been linked to preeclampsia. Reference intervals are usually based on healthy blood donors. Several physiological and immunological alterations occur during normal pregnancy with subsequent potential effects on blood analytes. Hence, this study aims to determine pregnancy-specific reference intervals for CRP and PTX3. Longitudinal clinical data and blood plasma samples from the 1^st^, 2^nd^ and 3^rd^ trimester of 100 healthy, non-medicating, females aged 18–40 at the time-point of conception were available to us. High‐sensitivity CRP measurements were performed by turbidimetry and enzyme-linked immunosorbent assay (ELISA) was used to quantify PTX3. CRP and PTX3 levels followed each other during the first two trimesters and both increased during the third trimester. CRP showed a median of 4.12 mg/L in the third trimester, and were significantly higher compared to the first (median 2.39 mg/L, p<0.0001) and the second (median 2.44 mg/L, p=0.0006) trimesters. In the third trimester PTX3 levels reached a median of 7.70 µg/L, and were significantly higher compared to the first (median 3.33 µg/L, p<0.0001) and the second (median 3.70 µg/L, p<0.0001) trimesters. Plasma albumin was inversely correlated with CRP (rho=-0.27, p<0.0001), but not with PTX3. In conclusion, it is important to consider pregnancy-specific reference values as elevations of CRP and PTX3 during the later phase may occur in absence of infection.

## Introduction

C-reactive protein (CRP) belongs to the evolutionary conserved pentraxin family. It is produced by hepatocytes in response to the inflammatory cytokines, mainly interleukin (IL) -6 and to a minor extent IL-1β, and its blood concentration may increase from less than 1 mg/L to 600–1000 mg/L (1). This rapid and profound increase makes CRP useful as a marker to monitor inflammatory activity in chronic diseases. Similarly, albumin in plasma may serve as a “negative” acute-phase protein (2). Circulating CRP levels are also used to distinguish bacterial from viral infections (1–3). By applying a cut-off of <10 mg/L, CRP may serve as a rough discriminator of bacterial from viral infections, since bacterial infections typically yield higher levels of circulating CRP. Viral infections are characterized by increased levels of interferon alpha, a cytokine shown to inhibit CRP synthesis (4, 5). By using high-sensitive technique and a different reference interval, CRP can reflect low-grade inflammation with clinical implications, e.g. cardiovascular diseases (6–8). Since 2010, high sensitivity CRP (hsCRP) detection has been used clinically as a biomarker for prognosis in patients with intermediate risk of cardiovascular disease.

The long pentraxin 3 (PTX3) is structurally and functionally related to CRP, but its production differs both with regard to its non-hepatic origin as well as to its inducing stimuli (9, 10). Monocyte and macrophage-derived production is induced by lipopolysaccharide (LPS) and IL-1β (11, 12), while the release of stored PTX3 from neutrophils is triggered by LPS and tumor necrosis factor (TNF) (11, 13). PTX3 is expressed by the cumulus cells, surrounding the oocyte, and actively participates in the organization of the matrix required for successful fertilization (14). Deficiency in PTX3 causes reduced fertility in mice and reduced litter size (14–17), aligning with data highlighting the importance of PTX3 for implantation (18). However, increased levels of PTX3 have also been associated with preeclampsia (19, 20). It is believed that there is an exaggerated inflammatory response and endothelial dysfunction in preeclamptic pregnancies, resulting in an increased release of inflammatory mediators such as PTX3 (21).

Reference intervals for CRP, and most other analytes, are routinely based on measurements of a large number of blood donors without ongoing medication or signs of inflammation. Pregnancy induces a number of physiological alterations and cannot directly be referred to a healthy female blood donor. The circulating blood volume as well as the blood viscosity undergo significant changes during childbearing. In the middle of the third trimester the blood volume has expanded up to 40% and the blood viscosity has decreased (22). Furthermore, considerable immunological alterations appear during pregnancy as the maternal immune system is confronted with foreign antigens derived from the semi-allogenic fetus and placenta (23). The immune system also changes during the developmental stages. The first trimester of pregnancy is pro-inflammatory to facilitate blastocyst implantation. The second trimester, a period of fetal growth, is characterized by an anti-inflammatory Th2 milieu (23). During the third trimester there is a switch towards a pro-inflammatory Th1 response which is necessary for nascency. Infections during pregnancies are common and CRP is frequently measured as a surrogate marker of infection. Studies investigating CRP and PTX3 levels longitudinal during pregnancy are scarce (24–28). Establishing population specific reference intervals is necessary for evaluating laboratory analytes. Hence, this study aims to determine pregnancy-specific serum CRP and PTX3 reference intervals.

## Materials and Methods

### Study Population

Characteristics of the participating women are outlined in **Table 1**. Plasma samples were available from a pregnancy biobank (Graviditetsbiobanken, GraBB), founded at the Linköping University in collaboration with University Hospital in Linköping. Longitudinal clinical data and EDTA-plasma samples from the 1^st^ (gestational week 8-11), 2^nd^ (gestational week 25) and 3^rd^ trimester (partus) of 100 healthy, without prescribed drugs, females aged 18–40 at the time-point of conception were available to us. None of the women were tobacco users and all had a body mass index (BMI) within the range of 18–25 kg/m^2^ at the time-point of conception. All were simplex pregnancies without pregnancy complications and the length of each pregnancy ranged from 37 + 0 to 41 + 6 weeks. Only women with a non-instrumental vaginal delivery and where the children had normal weight for gestational age were considered for the study. The women were also selected by date of birth, evenly distributed over quarters of the years to minimize impact of seasonal variations. The plasma samples were collected during the years 2015–2018 and had been frozen in –70°C until analysis.

**Table 1:** Characteristics of the participating women (*n*=100).

*Age, years (median, range)*	*30 (19–39)*
*BMI at inclusion, kg/m^2^ (median, range)*	22.1 (18.1–24.9)
*Gestation at delivery, weeks (range)*	40+1 (37+4–41+4)
*Multiparous women*	39 (39%)
*Birth period January–March*	25 (25%)
*Birth period April–June*	23 (23%)
*Birth period July–September*	27 (27%)
*Birth period October–December*	25 (25%)
*Fetal birth weight, gram (median, range)*	3565 (2510–4450)
*Fetal sex, female*	50 (50%)

### Detection of Plasma Albumin

Albumin (reference interval 34–45 g/L) was measured in plasma by turbidimetry at the Clinical Chemistry laboratory of the University Hospital in Linköping.

### Pentraxin Assays

CRP was measured with high‐sensitivity technique (detection limit 0.15 mg/L), performed by turbidimetry, at the Clinical Chemistry laboratory of the University Hospital in Linköping.

A DuoSet enzyme-linked immunosorbent assay (ELISA; detection limit 0.22 µg/L) kit was used to analyze PTX3 levels in plasma (R&D Systems, Minneapolis, MN, USA). Assays were performed according to the manufacturers’ instructions as previously described (29, 30). Briefly, Costar (Corning, NY, USA) half‐area plates were coated with 2 µg/mL of mouse anti‐human PTX3 and incubated overnight. Plates were blocked by 1% bovine serum albumin in PBS for 1 h and incubated thereafter with samples and standards for 2 h. Biotinylated goat anti‐human PTX3 (60 ng/mL) was added and incubated for 2 h, followed by addition of streptavidin horseradish peroxidase (R&D Systems) diluted 1:40 and another 20 min of incubation. Plates were developed with tetramethylbenzidine substrate and the reaction was stopped by adding 1 M H_2_SO_4_. All incubations were performed at room temperature. Plate reader VersaMax (Molecular devices, San Jose, CA, USA) and software SoftMax Pro version 5.4.1 (Molecular devices) were used.

### Statistical Analyses

Kruskal-Wallis tests were used to analyze differences in CRP, PTX3 and albumin levels during the three trimesters. The Mann-Whitney *U*-test was used to evaluate differences in PTX3 or CRP levels between primiparous and multiparous women and fetal sex. Spearman’s correlation was used to determine the association between pentraxins and BMI, fetal birth weight and fetal birth length. P-values <0.05 were considered significant. Statistical analyses were performed with SPSS statistics version 26 (IBM, Armonk, NY, USA) or GraphPad Prism version 9 (GraphPad Software, La Jolla, CA, USA). Undetectable levels of CRP were given half the value of the lower detection limit (*n*=7 out of 300). Levels of PTX3 above the range of detection were given 1.5 the value of the upper detection limit (*n*=4 out of 300).

### Ethics Statement

Oral and written informed consent was obtained from all participants during their first visit to the antenatal clinic, permitting biobank access to plasma samples as described above, and present and future medical records for research purpose. This information was given according to the Declaration of Helsinki. The study protocol was approved by the national ethical review agency (Decision number 2019-00424).

## Results

### CRP Levels During Pregnancy

**Figure 1:**
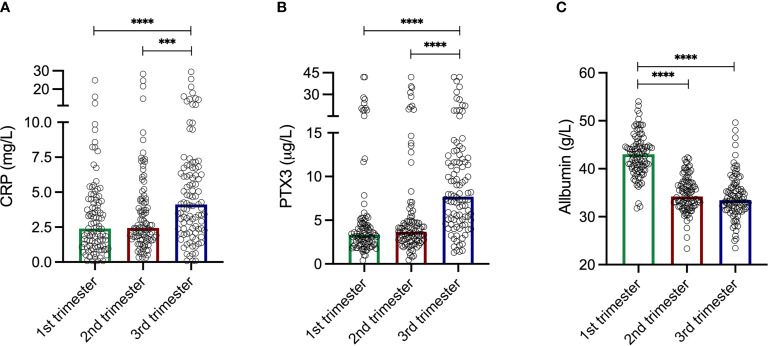
**(A)** C-reactive protein (CRP) levels during the three trimesters. First trimester median 2.39 mg/L. Second trimester median 2.44 mg/L. Third trimester median 4.12 mg/L. CRP levels increased during the third trimester and were significantly higher compared to the first trimester (p<0.0001) and the second trimester (p=0.0006). Please note axis break. **(B)** Pentraxin-3 (PTX3) levels during the three trimesters. First trimester median 3.33 µg/L. Second trimester median 3.69 µg/L. Third trimester median 7.70 µg/L. PTX3 levels increased during the third trimester and were significantly higher compared to the first trimester (p<0.0001) and the second trimester (p<0.0001). Please note axis break. **(C)** Plasma albumin levels during the three trimesters. First trimester median 43.02 g/L. Second trimester median 34.22 g/L. Third trimester median 33.46 g/L. The albumin levels were significantly higher compared to the second (p<0.0001) and the third (p<0.0001) trimester. Please note axis start. Kruskal-Wallis and Mann Whitney *U*-tests were used to analyze differences in CRP, PTX3 and albumin levels during the three trimesters. ***p = 0.0006, ****p < 0.0001.

The levels of CRP were comparable during the two first trimesters; median 2.39 mg/L in the first compared to 2.44 mg/L in the second (**Figure 1A**). CRP increased during the third trimester to a median of 4.12 mg/L, and were significantly higher compared to the first (p<0.0001) and the second (p=0.0006) trimesters. The CRP levels ranged from 0.08–24.8 mg/L, 0.3–28.3 mg/L and 0.08–29.4 mg/L in the first, second and third trimesters, respectively. Intervals for each trimester are shown in **Table 2**.

**Table 2:** Pregnancy-specific intervals for C-reactive protein (CRP, mg/L) and Pentraxin-3 (PTX3, µg/L).

	*Trimester 1*		*Trimester 2*		*Trimester 3*	
	CRP	PTX3	CRP	PTX3	CRP	PTX3
*Minimum*	0.08	0.40	0.30	0.44	0.08	1.29
*Maximum*	24.8	42.0	28.3	42.0	29.4	42.0
***Percentiles***						
*1st*	0.08	0.41	0.30	0.44	0.08	1.29
*3rd*	0.08	1.01	0.34	0.91	0.08	1.53
*5th*	0.30	1.52	0.56	1.30	0.56	1.97
*95th*	9.42	23.8	9.23	28.2	16.0	31.5
*97th*	12.2	27.8	21.4	34.5	21.2	39.1
*99th*	24.7	42.0	28.2	41.9	25.5	42.0

Significant weak correlations were found between BMI and CRP in the first (rho=0.21, p=0.03) and in the second (rho=0.23, p=0.02) trimester, but not in the third (rho=0.16, p=0.13). No significant differences in CRP levels during any of the trimesters were found between primiparous and multiparous women. Fetal sex did not affect the CRP levels during any of the trimesters. No significant correlations were found between CRP levels during any of the trimesters and fetal birth weight, nor between CRP levels during any of the trimesters and fetal birth length.

### PTX3 Levels During Pregnancy

Similar patterns as for CRP through the trimesters were found for PTX3. The first two trimesters showed comparable levels; median 3.33 µg/L in the first compared to 3.70 µg/L in the second (**Figure 1B**). During the third trimester the PTX3 levels increased to a median of 7.70 µg/L, and were significantly higher compared to the first (p<0.0001) and the second (p<0.0001) trimesters. PTX3 ranged from 0.40–42.0 µg/L, 0.44–42.0 µg/L and 1.29–42.0 µg/L in the first, second and third trimesters, respectively. Intervals for each trimester are presented in **Table 2**.

In contrast to CRP, PTX3 and BMI did not correlate in any of the trimesters. No significant differences in PTX3 levels during any of the trimesters were found between primiparous and multiparous women, and fetal sex did not affect the PTX3 levels during any of the trimesters. No significant correlations were found between PTX3 levels during any of the trimesters and fetal birth weight, nor between PTX3 levels during any of the trimesters and fetal birth length.

### Associations Between CRP, PTX3 and Albumin

**Figure 2:**
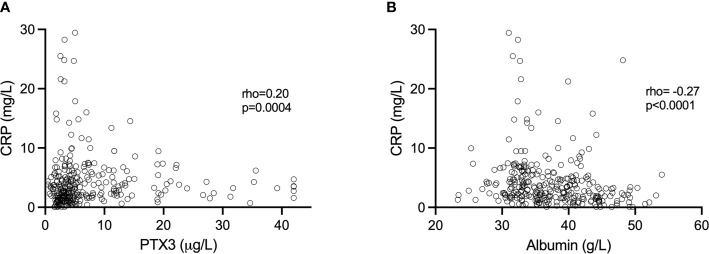
**(A)** Association of CRP and PTX3 values. **(B)** Association of CRP and plasma albumin values. Spearman’s correlation was used to determine the associations.

Altogether, when analyzing all samples, PTX3 was positively correlated with CRP (rho=0.20, p=0.0004; **Figure 2A**). However, we found no significant correlations between CRP and PTX3 when analyzing the three trimesters separately; first trimester (rho=0.10 p=0.31), second trimester (rho=0.10 p=0.32), third trimester (rho=0.01 p=0.96).

The albumin levels ranged from 31.76–54.02 g/L, 23.34–42.34 g/L and 23.46-49.62 g/L in the first, second and third trimesters, respectively (**Figure 1C**). Albumin was highest in the first trimester of pregnancy (median 43.02 g/L), and significantly higher compared to the second (median 34.22, p<0.0001) and the third (median 33.46 g/L, p<0.0001) trimesters. Collectively, when analyzing all samples, albumin was negatively correlated with CRP (rho=-0.27, p<0.0001; **Figure 2B**). Significant correlations were also found between albumin and CRP in the first (rho=-0.33, p=0.001) and in the second (rho=-0.23 p=0.02) trimester, but not in the third (rho=-0.002, p=0.98). No significant associations were found between PTX3 and albumin.

## Discussion

Although CRP is widely used as a surrogate marker of infection and inflammation in healthcare, specific reference intervals for pregnancies have not been established. This study was undertaken to define such reference intervals using data and samples from healthy Swedish women. In addition, we included PTX3 as increased levels have been proposed to associate with preeclampsia. To settle these kind of reference intervals are crucial to be able to evaluate any alterations from the normal response.

CRP has been reported elevated in pregnant compared to non-pregnant women (25, 31–33). In our study, CRP levels were similar in the first two trimesters and increased during the third trimester, which is in line with an older Swedish study by Larsson et al. (24), but in contrast to the recent observation by Dockree et al. (25). However, our cohort only consisted of women with a BMI within the range of 18–25 kg/m^2^ at the time-point of conception which was not the case in the study by Dockree et al. CRP has been shown to be further elevated in overweight and obese women and BMI-specific reference intervals has been proposed (25). The hepatic synthesis of CRP may be influenced by the amount of adipose tissue, through increased IL-6 secretion *in situ* (34, 35). In addition, CRP has been proposed to increase with BMI during pregnancy (25, 36), aligning our data reporting correlations, though weak, between CRP levels and BMI during the first and second trimesters. Altogether, both BMI and the gestational age should be considered as potential influencers. In comparison to Larsson et al. (24), where 15 of 52 (28.8%) women experienced minor complications, our cohort of pregnant women contained only healthy individuals and uncomplicated pregnancies. Contrary to our analyses, both Larsson et al. (26) and Yu et al. (27) performed their measurements in serum. Differences in absolute pentraxin levels between studies may be related to the use of serum *versus* plasma.

In contrast to CRP and in line with previous observations, the levels of plasma albumin were found to decrease with gestational age (33, 37). A significant inverse correlation was found between CRP and albumin, illustrating the divergent association of a “positive” and a “negative” acute-phase protein (2, 38).

In line with our results, the PTX3 levels has been reported to increase slightly with gestational age, with a more evident increase during the third trimester (26–28). Human chorionic gonadotropin (hCG), produced by the placenta, increases PTX3 expression by monocytic cells *in vivo* and *in vitro* (39). Moreover, hormones induced by hCG (*i.e.* progesterone and estrogen) may also stimulate production of PTX3. It has been suggested that the entire pregnancy period is characterized by raised PTX3 levels, that is first increased by hCG and subsequently by the hCG induced hormones (39). During the third trimester, the immune system alters to a more proinflammatory state essential for birth (23), which could be reflected by the increased levels of CRP and PTX3 at the late stage of pregnancy.

CRP and PTX3 levels showed a positive correlation in our study when pooled data were analyzed, confirming a previous report (27). However, when the trimesters were examined separately no significant associations were achieved. Thus, CRP and PTX3 cannot be interpreted as interchangeable analytes. CRP is produced by hepatocytes (1), whereas PTX3 is peripherally produced by non-hepatic cells such as monocytes/macrophages and neutrophils. Even though CRP and PTX3 are indeed structurally and functionally related, their production is additionally triggered by different inflammatory stimuli, which probably reflects potential of also diverging biological functions (1, 11–13). It has been demonstrated that the expression of PTX3 in the peripheral blood is closely related to preeclampsia (19), but no such pregnancy complications has yet been firmly established for CRP (32, 40–42).

The lack of pregnancy-specific reference intervals creates diagnostic uncertainty. Normal gestational variations of analytes are necessary to establish, otherwise normal physiological alterations during pregnancy may be misinterpreted as pathological. A proper reference interval may also restrict unnecessary interventions, such as unnecessary frequent maternal controls, prolonged antenatal hospital care or immobilization. A distinct reference interval could discriminate pathological pregnancies where inflammation already is a known important risk factor in an earlier stage than currently used such as preterm onset of labor or premature preterm rupture of membranes (43). It could also facilitate clinical decisions for managing complicated pregnancies such as preeclampsia.

Limitations should be acknowledged. Most women included were Caucasians, hence we cannot exclude possible differences between ethnic groups. In addition, it would have been convenient with an evaluation cohort to estimate the diagnostic accuracy of each analyte in women with infection and/or preeclampsia. Single nucleotide polymorphisms may indeed influence the blood levels of pentraxins; and it cannot be excluded that this could have affected the reference intervals presented (5, 44). Unfortunately however, in the present study we did not have ethical approval to perform DNA genotyping and make appropriate adjustments.

In conclusion, we report pregnancy specific reference values for the evolutionary highly conserved pentraxins CRP and PTX3 based on data from normal pregnancies in healthy women. Raised CRP and PTX3 levels may indicate infection and preeclampsia, respectively. Therefore, it is important to consider normal reference limits as both CRP and PTX3 tends to increase during pregnancy.

## Data Availability Statement

The original contributions presented in the study are included in the article/supplementary material. Further inquiries can be directed to the corresponding author.

## Ethics Statement

The studies involving human participants were reviewed and approved by the Swedish National Ethical Review Agency. The patients/participants provided their written informed consent to participate in this study.

## Author Contributions

LW: acquisition and analyses of patient data, interpretation of results and writing of the manuscript. SP, MS, and JW: interpretation of results and writing of the manuscript. CS: designing of the project, interpretation of results and writing of the manuscript. All authors contributed to the article and approved the submitted version.

## Funding

This work is supported by grants from Region Östergötland (ALF Grants), the Gustafsson Foundation, the King Gustaf V’s 80-year Anniversary Foundation and the King Gustaf V, Queen Victoria’s Freemasons’ Foundation, the Swedish Rheumatism Association and the Royal Swedish Academy of Sciences.

## Conflict of Interest

The authors declare that the research was conducted in the absence of any commercial or financial relationships that could be construed as a potential conflict of interest.

## Publisher’s Note

All claims expressed in this article are solely those of the authors and do not necessarily represent those of their affiliated organizations, or those of the publisher, the editors and the reviewers. Any product that may be evaluated in this article, or claim that may be made by its manufacturer, is not guaranteed or endorsed by the publisher.
